# An extensive dataset of handwritten central Kurdish isolated characters

**DOI:** 10.1016/j.dib.2021.107479

**Published:** 2021-10-14

**Authors:** Rebin M. Ahmed, Tarik A. Rashid, Polla Fatah, Abeer Alsadoon, Seyedali Mirjalili

**Affiliations:** a IT Department, Tishk International University, Erbil, Iraq; b Computer Science and Engineering Department, University of Kurdistan-Hawlêr, Erbil, Iraq; c Sallahadin University-Erbil, Erbil, Iraq; d School of Computer Data and Mathematical Sciences, Western Sydney University (WSU), Sydney, Australia; e Kent Institute Australia, Sydney, Australia; f Asia Pacific International College (APIC), Sydney, Australia; g Centre for Artificial Intelligence Research and Optimisation, Torrens University, Australia; h Yonsei Frontier Lab, Yonsei University, Seoul, South Korea

**Keywords:** Handwritten characters, Central Kurdish, Kurdish character recognition, Images of characters

## Abstract

To collect the handwritten format of separate Kurdish characters, each character has been printed on a grid of 14 × 9 of A4 paper. Each paper is filled with only one printed character so that the volunteers know what character should be written in each paper. Then each paper has been scanned, spliced, and cropped with a macro in photoshop to make sure the same process is applied for all characters. The grids of the characters have been filled mainly by volunteers of students from multiple universities in Erbil.

## Specifications Table

**Table:** 

Subject	Deep Learning
Specific subject area	Handwriting isolated character recognition of the Kurdish Language.
Type of data	Image
How data were acquired	Handwritten, Scanner, Marker
Data format	Raw Analyzed Filtered Jpeg image
Parameters for data collection	A form is designed to collect random copies of handwritten characters, The forms have been distributed among two main categories of volunteers: The academic staff and University students. All the letters were written in a black or dark blue pen on white paper.
Description of data collection	Each character was written on a printed grid to facilitate the operation of splitting characters from each other. Then they have been scanned with the same quality and split with the same methodology to ensure the exact size of each character file.
Data source location	Erbil/Erbil Governorate/Kurdistan Region Iraq
Data accessibility	An extensive dataset of Handwritten Central Kurdish Isolated characters. Data identification number: doi:10.17632/f8z9jts5nb.1 Direct URL to data: doi:http://dx.doi.org/10.17632/f8z9jts5nb.1

## Value of the Data

• The dataset is suitable for machine learning models for handwriting recognition.
• Researchers who have an interest in researches of Kurdish/Persian/Arabic language in deep learning and machine learning.
• This data can be a start for research of a more complex subject of joint characters and word recognition for this specific language.
• As it is highly standardized (meaning very carefully sized and formatted) it can be used as a benchmark of quality and usability for future works.

## 1 Data Description

Central Kurdish (*Sorani*) is one of two main dialects of the Kurdish language, it is generally thought that Sorani is spoken by about 9 to 10 million people in Iraq and Iran [1,2]. It is mainly written using a modified Arabic/Persian alphabet containing 34 characters, including characters that have been replaced in recent years like (ك) that's no longer been used by the Kurdish language and replaced with (ک). In this work, a comprehensive database has been created for isolated handwritten Central Kurdish character images containing 40,940 images with an average of 1170 images of each character written by 390 native writers. Table 1 shows the number of images and the Percentage of each character in the whole database. The repository in Mendeley^1^ consists of a samples folder that contains samples of each character, and a zip file containing the whole dataset that is described in this paper.

**Table 1:** number and percentage of the collected letters.

Order	ID	Letter	Number of images	Percentage
1	1	ئـ	1134	2.77%
2	2	ا	1134	2.77%
3	3	ب	1134	2.77%
4	4	پ	1008	2.46%
5	5	ت	1134	2.77%
6	6	ج	1134	2.77%
7	7	چ	1260	3.08%
8	8	ح	1260	3.08%
9	9	خ	1134	2.77%
10	10	د	1134	2.77%
11	11	ر	1134	2.77%
12	12	ڕ	1134	2.77%
13	13	ز	1512	3.69%
14	14	ژ	1123	2.74%
15	15	س	1107	2.70%
16	16	ش	1134	2.77%
17	17	ع	1260	3.08%
18	18	غ	1134	2.77%
19	19	ف	1134	2.77%
20	20	ڤ	1134	2.77%
21	21	ق	1260	3.08%
22	22	ک	1386	3.39%
23	23	ك	883	2.16%
24	24	گ	1134	2.77%
25	25	ل	1134	2.77%
26	26	ڵ	1134	2.77%
27	27	م	1386	3.39%
28	28	ن	1161	2.84%
29	29	هـ	1008	2.46%
30	30	ە	1512	3.69%
31	31	و	1134	2.77%
32	32	ۆ	1134	2.77%
33	33	وو	1134	2.77%
34	34	ی	1134	2.77%
35	35	ێ	1134	2.77%
	35		40940	100%

Although the Kurdish language uses modified Arabic/Persian (farsi) characters for writing, and there are many comprehensive databases of Arabic and Persian handwriting characters for offline character recognition and some databases even claim that their database can be used for recognition of other languages, such as Urdu and Kurdish [3,4]. However, there are two main problems, the first being that it does not contain all the characters used in Kurdish, like Re (ڕ), Ve (ڤ), Le(ڵ) and Wo (ۆ). The second problem is that it does not have consistency in the number and percent of characters that the Kurdish language uses.

## 2 Experimental Design, Materials, and Methods

### 2.1 Data collection

**Fig. 1:**
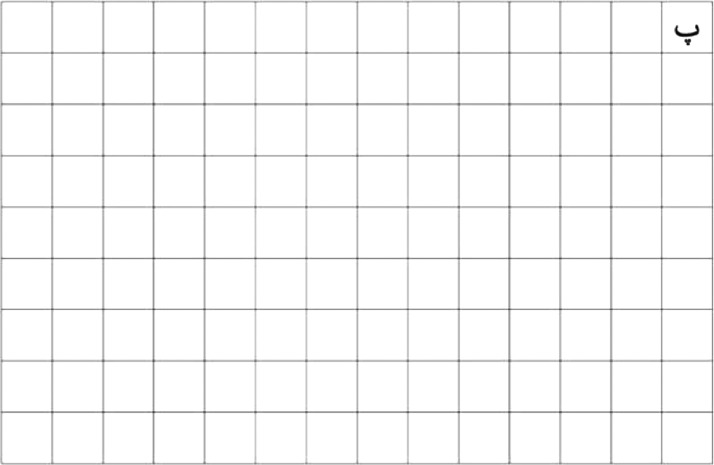
Form example.

Finding a suitable source of data is considered a first step toward building a database. Here, the main goal is to collect images of Kurdish handwritten characters written by many writers. So, a form is designed to do so. The form is shown in Fig. 1. It consists of 1 alphabet at a time letter that has been printed on the top right corner, and it has 125 empty blocks. The writers have been asked to write each letter three times in the three empty blocks. Thus, the total number of writers is 390.

The forms have been distributed among two main categories: The academic staff of the Information Technology department at Tishk International University, the university students of the University of Kurdistan-Hawler, Salahaddin University, and Tishk International University, As shown in Table 2. There were ten sets of forms, each set with 35 forms for 35 different letters; at first, we decided that nine sets, which will give us at least 1100 images for each letter, were the best option for the time that we had. However, then there were some problems with the collection process. In the first prints of the forms, there was confusion for instance, in Set 2, there were two forms for the letter (چ) and none for (ج), and since we printed and distributed the form at the same time, we were not aware of this problem until the stage of pre-processing, This was creating an inconsistency in the number of samples that we had, for example by the 9th set we had 504 images of the letter (ڤ), which was much less than other letters that they had at least 1000 images. So we decided to add the 10th set as a complementary to other sets, it only contained those letter, which was missing in the first nine forms, which was (ز،ژ،ش،غ،ڤ،ق،ک،ل،ن،ی), as explained in Table 3, the First column is the letter and columns 2-11 represent several images gathered in each set accordingly, while the first row the header row 2-36 are letters in each set, last row, and last columns are for the total of each letter and each set.

**Table 2:** Source of the data.

From	Number of Participants
Tishk International University - Students	300
Tishk International University - Staff	20
Salahaddin University - Students	60
University of Kurdistan-Hawler - Students	10

**Table 3:** Sets of data collection.

ID	Letter	Set 1	Set 2	Set 3	Set 4	Set 5	Set 6	Set 7	Set 8	Set 9	Set 10	Total
1	ئـ	126	126	126	126	126	126	126	126	126	0	1134
2	ا	126	126	126	126	126	126	126	126	126	0	1134
3	ب	126	126	126	126	126	126	126	126	126	0	1134
4	پ	126	126	126	126	126	126	0	126	126	0	1008
5	ت	126	126	126	126	126	126	126	126	126	0	1134
6	ج	126	0	126	126	252	126	126	126	126	0	1134
7	چ	126	252	126	126	126	126	126	126	126	0	1260
8	ح	126	126	126	126	252	126	126	126	126	0	1260
9	خ	126	126	126	126	126	126	126	126	126	0	1134
10	د	126	126	126	126	126	126	126	126	126	0	1134
11	ر	126	126	126	126	126	126	126	126	126	0	1134
12	ڕ	126	126	126	126	126	126	126	126	126	0	1134
13	ز	126	126	126	126	126	252	126	252	126	126	1512
14	ژ	126	0	126	126	126	0	0	126	126	367	1123
15	س	126	126	126	99	126	126	126	126	126	0	1107
16	ش	126	0	126	126	126	126	126	126	126	126	1134
17	ع	126	126	126	126	126	126	252	126	126	0	1260
18	غ	126	0	126	0	0	0	0	126	126	630	1134
19	ف	126	126	126	126	126	126	126	126	126	0	1134
20	ڤ	126	0	126	126	0	0	0	126	126	504	1134
21	ق	126	0	126	0	126	126	126	126	126	378	1260
22	ک	126	126	126	126	252	252	252	126	0	0	1386
23	ك	126	126	126	126	126	0	0	126	252	0	883
24	گ	126	126	126	126	126	126	126	126	126	0	1134
25	ل	126	0	126	126	0	0	0	126	126	504	1134
26	ڵ	126	126	126	126	126	126	126	126	126	0	1134
27	م	126	126	126	126	126	252	252	126	126	0	1386
28	ن	126	0	126	153	0	0	0	126	126	504	1161
29	ه	126	126	126	126	126	126	126	126	0	0	1008
30	ە	126	252	126	126	126	252	252	126	126	0	1512
31	و	126	126	126	126	126	126	126	126	126	0	1134
32	ۆ	126	126	126	126	126	126	126	126	126	0	1134
33	وو	126	126	126	126	126	126	126	126	126	0	1134
34	ی	126	0	126	126	0	0	0	126	126	504	1134
35	ێ	126	126	126	126	126	126	126	126	126	0	1134
	Total	4410	3528	4410	4158	4158	4031	3905	4536	4284	3520	40940

### 2.2 Form processing

**Fig. 2:**
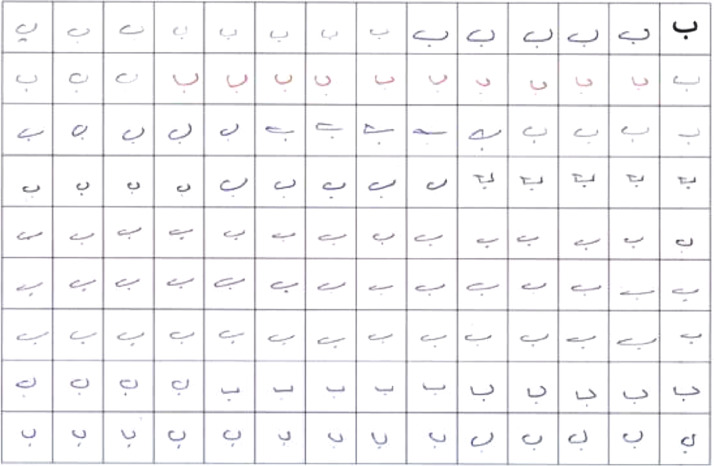
Scanned page example.

All form pages were scanned using a high-quality scanner. The scanner scans pages using 300 to 1800 dpi. The output of the scanner can be either a pdf, jpeg, bmp format. 600 dpi was used as it had more detail than 300 dpi and didn't make the file size as big as 1800 dpi, and the jpeg format was chosen because its compression makes it more suitable to store more than 40 thousand images. All the letters were written in a black or dark blue pen since the paper was white. An example of a scanned page is shown in Fig. 2.

**Fig. 3:**
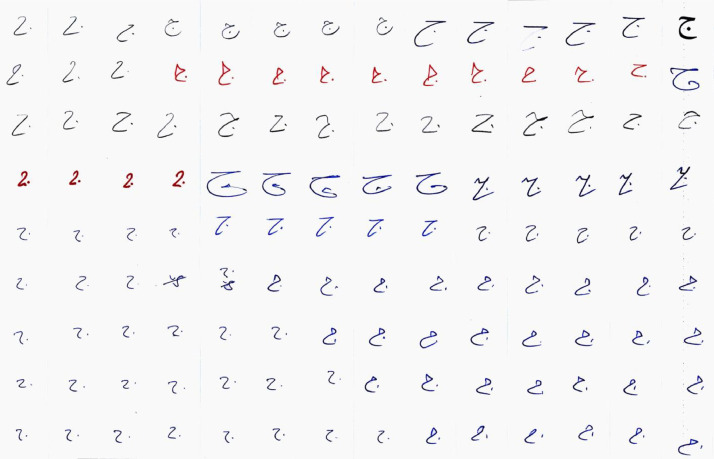
scanned page after pre-processing.

### 2.3 Pre-processing

The pre-processing phase is important in any recognition system. The goal of the preprocessing process is to improve the quality of the images for extracting the proper features later in any recognition system. A pre-processing process was applied to each form page to enhance the images. First of all, the Table border has been removed using the Eraser Tool in Adobe Photoshop software. The result of this step is shown in Fig. 3.

### 2.4 Cropping

**Fig. 4:**
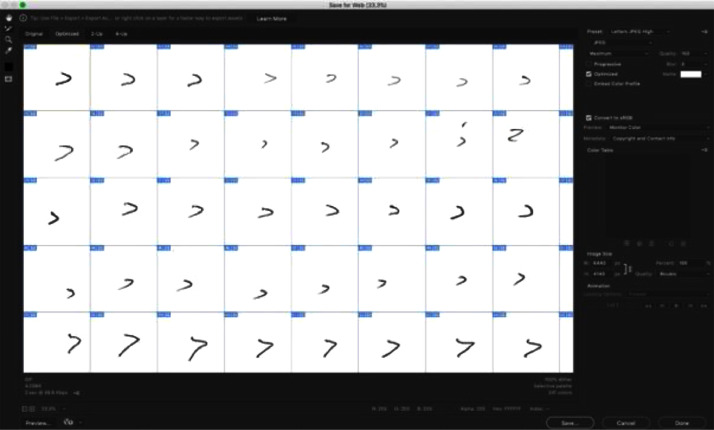
Slice tool in Adobe Photoshop software.

**Fig. 5:**
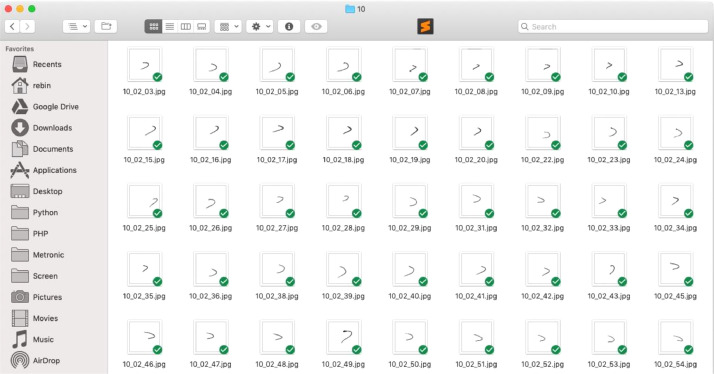
Result of slicing process.

After the pre-processing phase was completed, the cropping process was applied to each form page to crop each letter block. This process was done by designing a template using the Slice tool in Adobe Photoshop software. The template had a resolution of (6440 × 4140) pixels and divided the page into 9 rows and 14 columns, then cropped each letter, when saved templated generated 126 separate images of single characters from the page with the (460 × 460) pixels, Slice tool cropping, and the saving process is shown in Fig. 4, while the output of this process is summarized in Fig. 5.

In the process of cropping images, each letter was cropped and saved in a separate folder with the ID of the letter. The entire letter images were saved in the same size. Since each letter was written 125 times by 390 writers each writing three times resulting in 1050 images for each letter Table 3.

### 2.5 Labeling and organizing

Each image is labeled with three numbers and separated by an underscore, the first number is the id of the letter according to its position in the alphabetical order which is shown in Table 4, the second number being the number of the set of form which there was 10 sets each giving to a specific group of writers, the third number is the order of that character in the form which was between 1 to 126, so each image had a label like following 02_01_94.jpg, 02 is the id of the letter which in this case is Alef (١), then 01 being in the set number 1 which was given to 4th-grade students of Information Technology department in Tishk International University, and 94 is the order of that image in the form. Each letter was stored in a folder with its ID as the name of that folder, with each folder containing approximately 1134 images of that letter.

**Table 4:** Letter IDs.

ID	Letter	ID	Letter
1	ئـ	19	ف
2	ا	20	ڤ
3	ب	21	ق
4	پ	22	ک
5	ت	23	ك
6	ج	24	گ
7	چ	25	ل
8	ح	26	ڵ
9	خ	27	م
10	د	28	ن
11	ر	29	هـ
12	ڕ	30	ە
13	ز	31	و
14	ژ	32	ۆ
15	س	33	وو
16	ش	34	ی
17	ع	35	ێ
18	غ		

## Ethics Statement

All the handwritings were obtained with the consent of the individuals who had participated in the writing.

## CRediT authorship contribution statement

**Rebin M. Ahmed:** Data curation, Conceptualization, Methodology, Writing – review & editing. **Tarik A. Rashid:** Methodology, Supervision, Validation, Writing – review & editing. **Polla Fatah:** Methodology. **Abeer Alsadoon:** Supervision. **Seyedali Mirjalili:** Writing – review & editing.

## Declaration of Competing Interest

The authors declare that they have no known competing financial interests or personal relationships which have, or could be perceived to have, influenced the work reported in this article.

## Footnotes

1 https://data.mendeley.com/datasets/f8z9jts5nb/2.
